# Unrevealed roles of polyphosphate‐accumulating microorganisms

**DOI:** 10.1111/1751-7915.13730

**Published:** 2021-01-06

**Authors:** Ali Akbari, ZiJian Wang, Peisheng He, Dongqi Wang, Jangho Lee, IL Han, Guangyu Li, April Z. Gu

**Affiliations:** ^1^ School of Civil and Environmental Engineering Cornell University Ithaca NY 14853 USA; ^2^ State Key Laboratory of Eco‐hydraulics in Northwest Arid Region Xi’an University of Technology Xi’an Shaanxi 710048 China; ^3^ Department of Civil and Environmental Engineering Northeastern University 360 Huntington Avenue Boston MA 02115 USA

## Abstract

We first review current knowledge on PAOs, with a focus on bacteria, in terms of their phylogenetic identities, metabolic pathways and detection methods. We further discuss the evidence that suggests the ubiquitous presence of PAOs in nature and point out the unrevealed roles of the PAOs that warrant future investigation.

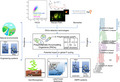

## Introduction

High‐polymeric inorganic polyphosphates (PolyPs) were first reported in living organisms by L. Liberman in 1890. The intracellular polyphosphates are polymers containing a few to several hundred tetrahedral phosphate residues linked through common oxygen atoms by phosphoanhydride bonds. They can adopt linear or cyclic ring structures. Monovalent or divalent metal elements, such as Mg^2+^, K^+^, Ca^2+^ and Na^+^ can serve as counterions in PolyP polymers, forming complexes with the negatively charged phosphate residues. PolyP occurs as a ubiquitous biopolymer in representatives of all kingdoms of living organisms and every cell type in nature. Recently, PolyP is coming into the spotlight because of the growing recognition of numerous cellular functions it performs. It plays roles in a wide range of microbial functions, including cellular phosphate and energy storage, the formation of membrane channels for active biomolecule uptake, cell envelope formation and function, cation storage and sequestration, pH buffering, enzyme activity regulation, stress responses, and survival and stationary phase adaptation (Kornberg, 1999; Rao *et al*., 2009).

The omnipresence of PolyP in natural environments is presumed to mostly originate from biotic processes involving organisms such as algae, fungi and bacteria. The phenomenon of intracellular PolyP storage in algae via either luxury P uptake or overcompensation has been long observed. PolyP accumulation in some fungal species has also been well documented in soil and marine environments. Both algae and fungi contribute to the PolyP pool in natural environments. In comparison, the presence and nature of PolyP‐accumulating bacteria in natural environments remain largely unexplored. Most of our knowledge of PolyP‐accumulating bacteria came from the extensive studies of engineered enhanced biological phosphorus removal (EBPR) systems for wastewater treatment. However, there is emerging evidence of the unexpected and ubiquitous presence of PolyP‐accumulating bacteria in natural environments such as rivers, lakes and soil, inviting endeavours to reveal their unknown functions and roles in the context of phosphorus availability and cycling.

In this article, we first review current knowledge on polyphosphate‐accumulating organisms (PAOs), with a focus on bacteria and their phylogenetic identities and metabolic pathways. We then examine genetic and phenotypic methods for PAO detection and their associated strength and limitations. We further discuss the evidence that suggests the ubiquitous presence of PAOs in nature and point out the potential roles of the PAOs in phosphorus storage and supply in the rhizosphere of soil, in eutrophication in water bodies and sediments, and in global carbon and nutrients cycling, which warrant future investigation.

## The ubiquitous presence of polyphosphate and PAOs in the environment

PolyP is likely ubiquitous and more abundant in nature than it has been recognized. PolyP has been detected using nuclear magnetic resonance (NMR) in lake sediments, marine settling particles and sediments, wetland soils and other terrestrial samples. For instance, PolyP was present in sediment samples from 18 out of 22 surveyed European lakes (Hupfer *et al*., 2004). PolyP’s ubiquity in nature has been mainly attributed to microbial activities, as abiotic generation requires extreme temperatures and pressures rarely seen in nature.

Bacterial cells with P‐rich inclusions were visualized by transmission electron microscopy (TEM) and energy‐dispersive X‐ray (EDX) in an environment associated with a high localized PolyP concentration (Hupfer *et al*., 1995). Microorganisms with intracellular P have been identified in the sediment, and PolyP formation and degradation was shown by ^33^P‐radiotracer and Raman spectroscopy (Goldhammer *et al*., 2010). Extensive PAO activity and high Candidatus *Accumulibacter* populations at levels comparable to those in EBPR systems were observed in an estuary sediment with a periodic change in dissolved oxygen (DO) condition (Watson *et al*., 2019). Fluctuating dissolved oxygen levels in estuaries offer ecological advantages for PAOs. Our preliminary investigation of the presence of PAOs in lake sediment implied the role of PAOs in the seasonal release of phosphorus from the sediment (Zhou *et al*., 2020). These observations provide preliminary indications about the significant role of PAOs in P cycling in the aquatic ecosystems.

In oligotrophic marine environments, PAOs such as *Beggiatoa* (Brock *et al*., 2012), diatoms and cyanobacteria accumulate intracellular PolyP as an energy reserve to adapt and survive environmental gradients or to scavenge nutrients. Also, PAOs attached to the settling particles can transport and actively exchange P with the environment through multiple water columns (Martin *et al*., 2014). Further, in the benthic zone, PAOs contribute to marine P burial and apatite formation (Ingall, 2010).

The existence of PAOs in the rhizosphere has been evidenced by the isolation of a PolyP‐accumulating strain of *Arthrobacter* (Li *et al*., 2013) and the detection of functional biomarker genes relevant to PolyP synthesis. Both polyphosphate kinase (*ppk*) and exopolyphosphatase (*ppx*) gene signals have been detected in maize croplands, and the intensities were significantly higher in the rhizosphere than in bulk soil (Li *et al*., 2014). In addition, higher PolyP detected in acidic wetland soils with higher organic matter contents implies PAO‐mediated C and P cycling in soil (Cheesman *et al*., 2014). We recently observed an enrichment of PAOs in maize rhizosphere soils as compared to bulk soils (unpublished). These observations suggest the potential involvement of PAOs in soil P cycling and their possible symbiosis with plant roots and crops.

## Missing PolyP in the global P cycling

Current quantitative P cycling models are based on the flux, retention time and transformation of the various forms of P between environmental compartments, and are mostly geochemically driven, with little inclusion of microbial processes. The models generally consider organic P in primary producers, terrestrial and marine biota, and those associated with various P minerals. The involvement of microbial activities in the P cycling models is generally limited to phosphorus‐solubilizing microorganisms and arbuscular mycorrhiza, which mediate the transformation of non‐bioavailable P into a bioavailable form.

The biological component of most global P cycling models is based on a presumed C/N/P ratio of cells, whereas PolyP storage and degradation alters the C/N/P ratio and influences the fate and transformation of P species that can be rather significant. PolyP‐to‐total phosphorus ratios have been reported as high as 27.1% in lake sediments (Ahlgren *et al*., 2011), 8% in marine (Sannigrahi and Ingall, 2005) and 30% in soil (Bedrock *et al*., 1994). PolyP was previously considered a non‐reactive P form; however, we now know that PolyP can be hydrolysed into reactive orthophosphate via spontaneous hydrolytic degradation under acidic conditions or via microbial enzymatic hydrolytic degradation. Because of the high abundance and potential reactivity of PolyP, its fate and transformation need to be included in a representative global P cycling model. However, the quantitative estimation of global PolyP dynamics is challenging. This is because the retention time of P in plants and animals is estimated to be on average 5–6 years, while the retention time of intracellular PolyP in microorganisms is only several hours and varies with environmental conditions. The time and spatial scales at which the PolyP is relevant need further investigation.

## Current knowledge of PAOs

PolyP accumulation in diverse members of almost all classes of algae, including members of *Chlorophyceae* (green algae), *Phaeophyceae* (brown algae) and *Rhodophyceae* (red algae), has been reported since the 60s (Cembella *et al*., 2008). Similarly, PolyP accumulation in various fungi, including members of *Mucoromycota,* particularly in relation to plant symbiosis, is well characterized (Ezawa *et al*., 2004). In comparison, limited bacterial PAOs have been identified to date, which include members of Proteobacteria (*Candidatus* Accumulibacter*, Dechloromonas*, *Pseudomonas*, *Ca*. Accumulimonas, Quatrionicoccus, *Malikia*, *Lampropedia*), Actinobacteria (*Tetrasphaera*, *Microlunatus*, *Tessaracoccus*, *Candidatus* Microthrix, *Friedmaniella*) and Gemmatimonadetes (*Gemmatimonas*). *Ca*. Accumulibacter and *Tetrasphaera* are two major lineages that have gained particular attention and are frequently identified in EBPR systems in geographically distinct full‐scale wastewater plants, including in Denmark, USA and Australia (Beer *et al*., 2006; Nielsen *et al*., 2019; Onnis‐Hayden *et al*., 2020). The adaptation capacity of PAO communities is reflected in the relatively higher spatiotemporal stability of PAO communities compared with overall communities in wastewater plants. Fine‐resolution microdiversities have been characterized by functional gene analysis and indicated distinct clades within each lineage with distinct physiological properties (Mao *et al*., 2015). PAO clades were correlated with operational parameters and influent properties, but not geographic locations. Similar observations have been reported in natural systems such as sediments. These observations indicate the significance of ecological selection in shaping PAO communities.

Most of our knowledge about the physiology of PAOs originates from studies with *Ca*. Accumulibacter phosphatis, the dominant agent in EBPR systems that select PAOs via an alternating carbon‐rich anaerobic phase and aerobic/anoxic phase. Accumulibacter‐like PAOs possess a unique and versatile carbon and energy management system via the adaptive utilization of different combinations of three intracellular polymers, namely PolyP, glycogen and polyhydroxyalkanoate (PHA). Both PolyP and glycogen can serve as energy sources for volatile fatty acid (VFA) uptake and PHA synthesis under the anaerobic regime, and then, they are replenished during the PAO’s obligate aerobic or anoxic growth, using PHA as the carbon source. Besides glycolysis from glycogen, the reducing power requirement in anaerobic PHA synthesis is also balanced by the versatile TCA cycle operation patterns. The versatile TCA operation patterns and regulation mechanisms are among the least understood and most debated PAO metabolism aspects. This metabolic versatility allows Accumulibacter PAOs to adapt to and gain a competitive advantage under a wide range of substrate composition and intracellular polymer availability conditions while maintaining their PHA accumulation activity. Using single‐cell Raman microspectroscopy and agent‐based modelling, we recently confirmed others’ observations (Zhou *et al*., 2009; da Silva *et al*., 2018) and provided cellular‐level evidences for the adaptive metabolic pathways in PAOs, who can generate the energy needed for cell maintenance and decay depending on the availability of different intracellular polymers (PolyP and glycogen) via glycolysis pathway and/or alternative TCA cycle operations (Li *et al*., 2018b, 2020; Majed and Gu, 2020). A completely different metabolic pattern for the novel PAO *Tetrasphaera* was recently demonstrated (Kristiansen *et al*., 2013). *Tetrasphaera* possesses a fermentative metabolism, utilizes sugars and amino acids, and performs PolyP accumulation without PHA accumulation. These variations in the metabolic properties of PAOs likely offer different ecological niches for different lineages.

## Challenges in PAO detection and quantification

Our current phylogenetic understanding of PAOs relies on previous 16S rRNA gene amplicon sequencing, fluorescence in situ hybridization (FISH), metagenomics and functional genomics analyses. Sequencing the conserved regions of polyphosphate kinase (*ppk*) and exopolyphosphatase genes has been used to delineate higher‐level phylogenetic (micro)diversity of PAOs beyond that offered by 16S rRNA sequencing, and to identify clades within each PAO lineage (McMahon *et al*., 2007). However, correlations between functional gene clades and the extent and mechanism of P removal are yet to be established. The incongruencies between *ppk* clades and 16S rRNA phylogenetic clusters could be used for tracing horizontal gene transfer networks. Comparative FISH studies with *ppk* probes versus PAO general probes, and the current repository of *ppk*‐harbouring taxa, indicate a significantly higher diversity of PAOs than previously known from EBPR systems. The identification of those unknown potential PAOs detected with *ppk* probes could be the subject of further studies. Methods such as high‐throughput epicPCR (emulsion, paired isolation and concatenation PCR) can directly identify the functional gene‐harbouring taxa (Spencer *et al*., 2016). Metagenomic approaches could also be used to characterize the metabolic potentials and functional diversity of PAOs in engineered or natural systems, and that could be significantly empowered with reference genomes from single‐cell sequencing of PAOs. A combination of genome‐based metagenomic and *in situ* transcriptomics paves the way for unravelling genome–activity relationships and developing predicting models.

Various approaches have been employed for phenotypic detection and fingerprinting of PAOs, and most of them are based on PolyP granule identification. Comprehensive reviews of PolyP detection methods have been published by Majed *et al*. (2012) and Wang *et al*. (2020). The staining of PolyP with fluorescent dyes is a straightforward but relatively imprecise technique and has difficulties quantifying the dynamics of PolyP in each cell. Raman spectroscopy, on the other hand, can provide quantitative information on intracellular PolyP at the single‐cell level resolution (Li *et al*., 2018a). FISH‐based flow cytometry, Raman–FISH and microautoradiography combined with FISH (MAR‐FISH) have been used for phenotypic analysis of targeted PAOs (Fernando *et al*., 2019). FISH requires prior knowledge of target taxa; therefore, these methods are limited to analysis of known PAOs. Fluorescence‐activated cell sorting (FACS) and Raman‐activated cell sorting (RACS) could be used to separate PAO cells, followed by downstream sequencing of sorted cells to characterize their phylogenetic diversity. The throughput of RACS is limited due to the required scanning time and complexity of PolyP Raman bands. A combination of stable isotope probing and Raman (Raman–SIP) offers a straightforward technique for identifying target chemical bonds. Labelling of chemical bonds leads to a distinct, easily identifiable shift in their Raman band. In the context of microbial ecology, labelling with D_2_O, ^13^C and ^15^N has been used to identify metabolically active cells, metabolic versatility and nitrogen uptake respectively. ^18^O‐labelled phosphate has been used for mass spectrometry analysis of RNA molecules. It is conceivable that similar labelling approaches could enhance the identification of labelled intracellular PolyP in PAOs. Such approaches combined with technological advancements in vibrational spectroscopy could be used for high‐throughput functional analysis of PAOs in complex microbial communities. An integrated platform of Raman and cell manipulation can be further used to isolate single cells with the desired trait, such as phosphate accumulation. Isolated single cells can then be sequenced or, given that the technique is non‐destructive, ideally cultivated. Single‐cell genomes of diverse PAOs is a significant gap in our current knowledge of PAOs. Additionally, such single‐cell level methods offer the opportunity to address the functional heterogeneity within each taxon, which has significant implications in overall population function, but is mainly unexplored due to technological limitations.

## Future outlook

The frequent detection of PolyP with the biological origin at relatively high abundances indicates the significance of PAOs in the P cycle. The preliminary genomic or physiological evidence suggests that major PAOs such as *Ca*. Accumulibacter and *Tetrasphaera* can potentially perform partial or complete denitrification. Also, PAOs have different metabolic preferences, and some can accumulate PHA. Glycogen and polyhydroxybutyrate storage and degradation in the important nitrogen‐fixing *Rhizobium leguminosarum* bacteria are known to fuel bacteroid differentiation (Xu *et al*., 2017). The *Rhizobium leguminosarum* is a putative PAO harbouring *ppk*, and thus, it is likely that PolyP storage and degradation similarly contribute to the symbiotic nitrogen fixation process. Therefore, beyond the plausible role in the P cycle, PAOs’ activity may have significant impacts on natural C and N cycles.

In the rhizosphere, there is a dynamic P exchange regulated by plants, microorganisms, and abiotic processes involving the solubilization, mobilization and uptake of P. Temporal redox fluctuations at transient zones or wetting–drying cycles from precipitation or irrigation may offer an ecological advantage for PAOs. PAOs could serve as a sink for P in the unsaturated vadose zone. On the other hand, they release bioavailable inorganic P under anoxic conditions, which can occur under saturated conditions from irrigation. Practically, therefore, they can serve as a controlled release P supplier.

The availability of P controls microbial activity in lakes, as both carbon and nitrogen can be microbially fixed from the atmosphere. Therefore, the understanding of P loading and cycling is essential in limnology and eutrophication. PAOs generate PolyP via luxury uptake from sediments under oxic conditions when surplus P is available. Heterotrophs and PAOs in the anaerobic hypolimnion contribute to internal loading by releasing the PolyP and organic P from sediments. Such a microbially mediated P cycle continuously supplies phosphorus in the environment and supports sustained long‐term microbial activity, even without external loading. The ecological selection of PAOs may occur at the interface of hypolimnion and epilimnion with oscillating oxygen levels or, for example, during fall mixing. Under eutrophic conditions, targeted biostimulation of benign PAOs, as a remedy, may reduce the availability of P for cyanobacteria in the oxic epilimnion, and limit their growth and associated ecological damages. Comparative analysis of wastewater treatment plants and eutrophic lakes provides insights on the ecological drivers of microbial community assembly and PAOs in P‐rich environments, for ultimately harnessing their potential for biotechnological applications and improving water quality.

Despite our rapidly evolving knowledge about PAOs, including the recent metabolic reconstructions of the well‐known PAOs from their genomes, their cellular‐level biochemical mechanisms remain largely uncharacterized. The yet to be explored relationships between the functional gene clades of PAOs and their traits are also essential for developing predictive models of the ecosystem. The effects of environmental factors at various scales and climate‐induced changes on the ecological selection of PAOs, and their physiology are also unknown.

Most of the previous studies in the context of engineered EBPR systems have focused on a reductionist approach and are limited to the analysis of a few known PAOs for the sake of process optimization. However, intracommunity interactions likely contribute to observed PAO behaviour under various environmental or operational conditions. Therefore, holistic microbiology, ecology and geochemistry approaches are needed to delineate the unrevealed role of PAOs in the environment.
